# Experimental demonstration of integrated encryption and communication over optical fiber

**DOI:** 10.1093/nsr/nwaf112

**Published:** 2025-04-02

**Authors:** Zekun Niu, Yunhao Xie, Guozhi Xu, Chenhao Dai, Hang Yang, Chuyan Zeng, Minghui Shi, Lyv Li, Guoqing Pu, Weisheng Hu, Lilin Yi

**Affiliations:** Department of Electronic Engineering, State Key Laboratory of Photonics and Communications, School of Electronic Information and Electrical Engineering, Shanghai Jiao Tong University, Shanghai 200240, China; Department of Electronic Engineering, State Key Laboratory of Photonics and Communications, School of Electronic Information and Electrical Engineering, Shanghai Jiao Tong University, Shanghai 200240, China; Department of Electronic Engineering, State Key Laboratory of Photonics and Communications, School of Electronic Information and Electrical Engineering, Shanghai Jiao Tong University, Shanghai 200240, China; Department of Electronic Engineering, State Key Laboratory of Photonics and Communications, School of Electronic Information and Electrical Engineering, Shanghai Jiao Tong University, Shanghai 200240, China; Department of Electronic Engineering, State Key Laboratory of Photonics and Communications, School of Electronic Information and Electrical Engineering, Shanghai Jiao Tong University, Shanghai 200240, China; Department of Electronic Engineering, State Key Laboratory of Photonics and Communications, School of Electronic Information and Electrical Engineering, Shanghai Jiao Tong University, Shanghai 200240, China; Department of Electronic Engineering, State Key Laboratory of Photonics and Communications, School of Electronic Information and Electrical Engineering, Shanghai Jiao Tong University, Shanghai 200240, China; Department of Electronic Engineering, State Key Laboratory of Photonics and Communications, School of Electronic Information and Electrical Engineering, Shanghai Jiao Tong University, Shanghai 200240, China; Department of Electronic Engineering, State Key Laboratory of Photonics and Communications, School of Electronic Information and Electrical Engineering, Shanghai Jiao Tong University, Shanghai 200240, China; Department of Electronic Engineering, State Key Laboratory of Photonics and Communications, School of Electronic Information and Electrical Engineering, Shanghai Jiao Tong University, Shanghai 200240, China; Department of Electronic Engineering, State Key Laboratory of Photonics and Communications, School of Electronic Information and Electrical Engineering, Shanghai Jiao Tong University, Shanghai 200240, China

**Keywords:** security communications, optical fiber communications, deep learning, mutual information, integrated encryption and communication

## Abstract

As we enter the big data and artificial intelligence (AI) era, integrating security and communication over optical fiber has become a critical challenge. This urgency is driven by the need to protect vast amounts of sensitive data, ensuring privacy security across global high-capacity optical networks. Traditional secure communication methods often struggle to maintain high-capacity transmission performance while providing robust security. Here we propose an integrated encryption and communication (IEAC) framework, designed to maximize mutual information (MI) for legal users while minimizing it for potential eavesdroppers. Enabled by end-to-end deep learning, this holistic framework trains a random number-selected geometric constellation shaping scheme to optimize encryption processes and transmission quality simultaneously. The IEAC experiment system achieves a groundbreaking single-channel transmission rate of 1 Terabit per second (Tb/s) over a 1200-km fiber link, employing a 26-channel, 3.9 THz bandwidth, full C-band wavelength division multiplexing (WDM) configuration. The MI for eavesdroppers is under 0.2 bit per symbol where the regular value is near 4.0, ensuring secure transmission. The IEAC scheme offers a scalable, promising solution to meet the escalating demand for high-throughput, secure data transmission in the face of advancing big data and AI computational technologies.

## Teaser

Researchers propose an integrated encryption and communication framework via end-to-end deep learning. They demonstrate a 1 Tb/s secure optical transmission experiment over a 1200 km fiber link.

## INTRODUCTION

In the big data and artificial intelligence (AI) era, the sheer quantity of sensitive information being handled, ranging from personal data to critical infrastructure information, necessitates robust encryption against unauthorized access [1–3]. The physical layer, such as the optical fiber channel, is vulnerable to various tapping attacks, including residual adjacent channel crosstalk listening [4–6]. Furthermore, emerging technologies, particularly machine learning, pose a threat by potentially weakening the security of existing cryptographic methods [7]. Additionally, physical layer security should be seen as complementary to higher-layer security mechanisms, providing an extra layer of protection against adversaries who may bypass or compromise upper-layer protocols. Therefore, an integrated encryption and communication (IEAC) system is crucial for securing the physical layer [8]. This ensures high-capacity, secure data transmission in both optical fiber and wireless channels, thereby preserving privacy and preventing catastrophic data breaches in public networks [9].

Given the massive volumes of data requiring real-time processing and secure communication from data centers to support cloud services, high-capacity optical fiber long-haul transmission technologies have emerged as crucial for fulfilling these demands, thus facilitating ongoing advancements in AI and big data areas. While the capacity of optical fiber transmission has surged to 800 Gbps per wavelength over 1200 km, with the potential to reach 1 Tb/s in the future [10–12], traditional secure communication systems lag behind, typically operating under 400 Gbps per wavelength for long-haul transmissions extending beyond 1000 km [13–15]. Existing methodologies like quantum key distribution (QKD), chaotic optical communication, and quantum noise stream ciphers (QNSC) provide robust security but at the cost of communication performance. QKD, while theoretically secure, typically offers a key rate of few megabits per second. The highest record of QKD is ∼115.8 Mbps over 10 km standard optical fiber [16–20]. This limitation is primarily due to fiber loss and the intrinsic capacity constraints of the quantum channel, making it less feasible for high-capacity transmission [21]. Chaotic communication systems suffer from performance degradation due to chaotic synchronization issues for the long-haul optical fiber transmission with the highest record of 256 Gbps transmission over 1600 km fiber [15,22–26]. QNSC manifests heightened sensitivity to laser noise, thereby compromising its reliability in typical coherent optical fiber communication scenarios, capping at 201.6 Gbps over a distance of 1200 km [14,27–29]. These limitations arise because traditional security frameworks in communications often concentrate on embedding robust security mechanisms into the communication process [13–15]. They present a lack of design from an integrated perspective that considers both communication and encryption as a unified entity, thus resulting in the trade-off between security and communication.

Against this backdrop, our research introduces an IEAC paradigm to achieve robust security of optical fiber communications while simultaneously maintaining its high communication performance. We design an IEAC framework, optimizing mutual information (MI) for legitimate users and minimizing it for illegal ones. Leveraging the end-to-end deep learning (E2EDL), a dynamic geometric constellation shaping (GCS) using high-speed digital random numbers is achieved in order to guarantee each symbol refers to different GCS mapping, showing no performance degradation in the additive white noise (AWGN) channel. Experimentally, we achieve a record-breaking single-channel secure transmission rate of 1 Terabit per second (Tb/s) over a 1200-km optical fiber link, while simultaneously utilizing 26 wavelength division multiplexing (WDM) channels to fully span the C-band bandwidth of 3.9 THz. The proposed scheme demonstrates robust security by maintaining the MI for an illegal (unauthorized) user at a level below 0.2 bits per symbol in experiments. This milestone not only showcases the potential of deep learning to revolutionize optical fiber communications but also marks our solution as a scalable, avant-garde response to the burgeoning IEAC demand for big data and AI computational technologies.

## INTEGRATED ENCRYPTION AND COMMUNICATION FRAMEWORK

**Figure 1.**
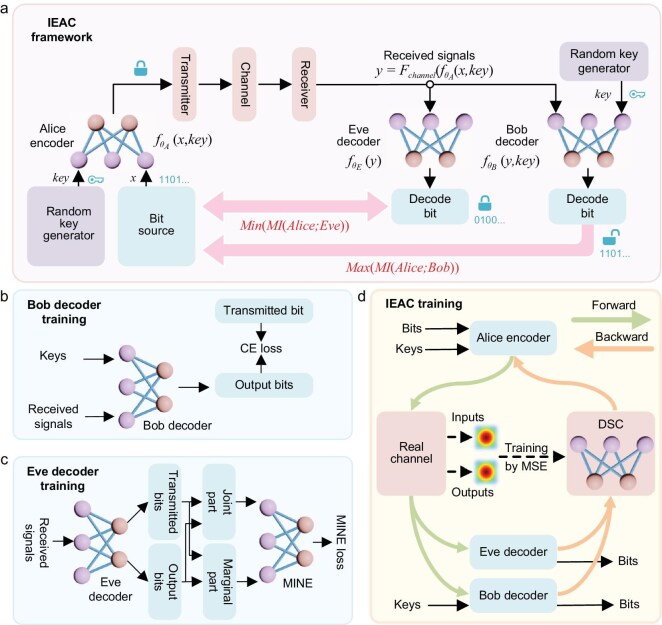
The IEAC framework. (a) The overall architecture of IEAC, where three NNs—one encoder and two decoders—collaborate to maximize MI between Alice and Bob (legal user with key) while minimizing MI between Alice and Eve (illegal user without key). (b) Training of Bob decoder using CE loss. (c) Training of Eve decoder employing MINE loss. (d) Training of the encoder based on gradient backpropagation. Throughout the training process, the forward path (green line) proceeds through the real channel, while the backward path (orange line) utilizes the DSC, which is itself trained using real channel data with MSE loss.

The core principle of IEAC is optimizing MI—maximizing it for legal users who have the key and minimizing it for illegal users without the key. This objective is realized through a comprehensive E2EDL, which is meticulously designed to optimize the entirety of the encryption and communication process, as illustrated in Fig. 1a. Predicated on the premise of an ideal random key distribution accessible to legal users, the encryption is managed within the transmitter Alice encoder neural network (NN) with parameter θ*_A_*, which is the pivotal element for optimization via E2EDL. The E2EDL also operates with two decoders, denoted as Bob decoder with parameter θ*_B_* and Eve decoder with parameter θ*_E_*. The main distinction between these decoders lies in access to the encryption key: one operates with the key, emulating a legal receiver, while the other without it, simulating an eavesdropper's attempt to decode the message illegally.

For legal users, optimization is based on the Barber–Agakov (BA) estimation method [30,31], leveraging Bob decoder to minimize the well-known cross-entropy (CE) loss at the receiver, as shown in Fig. 1b, thereby theoretically maximizing the MI [32–34]. Conversely, for illegal users, MI is initially estimated using the enhanced Mutual Information Neural Estimation (MINE) technique, as illustrated in Fig. 1c, which employs the Donsker-Varadhan (DV) dual representation [35,36]. MINE can estimate the MI without channel models and can be extended to the optical fiber channels [36]. Following this estimation, MI minimization for illegal users is executed leveraging MINE (see Supplementary Data for details). Employing *x* and *y* to symbolize the ordinary signal before the transmitter encoder and receiver decoder, respectively, then the E2EDL loss function for the IEAC framework can be encapsulated by:


(1) \begin{eqnarray*}
Los{s_{{IEAC}}} &=& Los{s_{CE}}(x,{f_{{\theta _B}}}(y,key))\\
&& +\, \textit{Loss}{ _{{MINE}}}(x,{f_{{\theta _E}}}(y)),
\end{eqnarray*}

where *f_θ_*(∙) represent the decoder NN function with parameter *θ*. The received signal *y* is a function of encoded signal:


(2) \begin{eqnarray*}
y = {F_{{channel}}}({f_{{\theta _A}}}(x,key)),
\end{eqnarray*}

where *F_channel_*(∙) is a channel transfer function. By applying E2EDL throughout the entire communication process, from signal encoding, transmission to reception, we ensure a holistic optimization of the system's IEAC performance. In essence, the E2EDL enabled IEAC framework offers a sophisticated, dual-faceted strategy: it not only refines MI optimization for authorized (legal) communication but also simultaneously restricts potential information leakage to unauthorized (illegal) parties. The encoder is a fully-connected neural network (FCNN) with 3 hidden layers (256 neurons each), optimized for symbol mapping. The activation function is the ReLU function. The decoder NN has the same structure as the encoder with residual connections to mitigate gradient vanishing. Note that the Eve decoder *θ_E_* is not assumed to represent a real-world eavesdropper's parameters during deployment. Instead, it acts as a simulated adversary during the E2EDL training phase. By training the encoder to minimize MI against a diverse set of simulated eavesdroppers (parameterized by *θ_E_*), we enforce robustness against a broad class of potential attackers. This adversarial training paradigm is analogous to generative adversarial networks (GANs) [37], where a generator (encoder) learns to fool a discriminator (Eve decoder) without requiring prior knowledge of real-world adversaries.

In practice, the E2EDL-based IEAC framework is constructed from several NN models trained to perform specific tasks, including encoder, channel model, decoder and MINE. These models are trained on the optical fiber channel with a vast amount of data, ensuring the encoded signal after encryption learned to recognize and adapt to a variety of distortions typical in long-haul optical fiber communication. Due to an unknown channel mathematical model, the E2EDL is challenging through backpropagation [34,38–40]. To achieve stable training, a dual-path training is employed with a differential surrogate channel (DSC) approach, as demonstrated in Fig. 1d: the forward path uses the real channel, while the backward path utilizes the DSC to facilitate the backpropagation of gradient error in Eq. (2). The DSC is trained as a regression task through a Mean Square Error (MSE) loss function, aiming to establish an accurate model of the channel's behavior [41,42]. This dual-path strategy is pivotal for accurately simulating the transmission environment during the training phase, thereby optimizing the encoder NN performance in actual communication scenarios. Furthermore, we also use a step-by-step training strategy, which will be introduced in the Supplementary file, enabling the encoder to achieve optimal performance with multiple NN collaborations under real channel scenarios.

The proposed E2EDL-based IEAC framework facilitates an integration of security mechanisms directly into the communication process. By doing so, it eliminates the need for separate, often cumbersome, security systems that might significantly degrade the performance of data transmission. Instead, security becomes an intrinsic, indistinguishable part of the communication flow, ensuring high-speed and long-distance transmission without sacrificing security.

## GEOMETRIC CONSTELLATION SHAPING ENABLED ONE-TIME PAD-LIKE ENCRYPTION

Within our E2EDL implementation, we employ GCS, an efficient technique that modulates signal constellation points within the complex plane [43–45] to achieve the IEAC framework. In this case, the encoder of the E2EDL is trained and outputs the suitable GCS map according to the input key. For each symbol transmission, the constellation map undergoes alterations based on the key derived from a digital number and the transmitted symbol is selected by bits, which are generated from either uniform or Gaussian random distribution. By coupling GCS within the E2EDL frameworks, the one-time pad-like encryption method—known for its theoretical uncrack ability—is achieved.

The security key of our approach utilizes a random noise sequence rather than conventional random digital bits. This sequence can be derived from either a high-speed digital number generator or a chaos sequence generator. Specifically, the method employed in this study generates Gaussian noise via the Permuted Congruential Generator (PCG) algorithm in NumPy with Python [46]. In the context of our training methodology, a 64-bit seed key is used to generate a key stream with a period of 2^64^–1. To maintain security, same as the QNSC methodology, the seed of the random generator requires periodic key distribution, a process that can be integrated with QKD schemes [16–20]. In this case, the utilization of a unique GCS for each transmission, dictated by the randomness of the selected digital numbers, significantly enhances the security of IEAC, rendering information theft exceedingly challenging for illegal users to decode in the absence of the precise key. This stochasticity guarantees security without relying on precise knowledge of *θ_E_* post-training.

**Figure 2.**
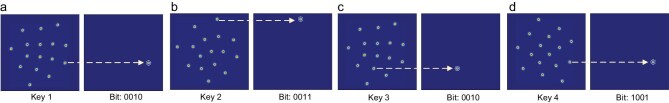
IEAC GCS examples. (a) An initial GCS map (left) serves as a comparative reference, juxtaposed with a transmitted symbol according to input bits 0010 (right). (b–d) Sequentially showcasing rotation (b), coding (c), and positional adjustments (d) in GCS maps according to the key, each pair comprising the altered map (left) and its corresponding transmitted symbol with input bits (right).

Within our framework, the trained GCS scheme is distinguished by three core manipulations: rotation, coding, and position. Figure 2a employs a GCS map as a comparative benchmark. The first manipulation entails key-dependent rotation of the constellation diagram, altering its orientation in the complex plane, as depicted in Fig. 2b. The second manipulation, illustrated in Fig. 2c, modifies the encoding of constellation points while preserving Gray encoding for optimal bit error rate (BER) performance. The final strategy, exemplified in Fig. 2d, adjusts the geometric arrangement of constellation points based on the different key. Each of these manipulations introduce a layer of security by complicating the signal's characteristics, making it increasingly difficult for illegal entities to interpret the transmission without compromising the system's overall communication performance.

Furthermore, the one-time pad-like ensures that the set of transmitted symbols, when viewed without the corresponding key, resembles random noise, as illustrated in Fig. 2a. With different input bits and random keys, the transmitted symbols fill the entire complex plane. Figure 2b shows the encryption signal's distribution, which exhibits characteristics of probabilistic shaping. This distinctive pattern arises because, in GCS, the signal density typically decreases towards the outer boundaries while concentrating towards the central area. Incorporating one-time pad-like encryption into this setup results in an abreast signal distribution that closely mirrors a Gaussian distribution. This Gaussian-like alignment optimizes Euclidean distance under power constraints, contributing not only to enhanced transmission performance but also guaranteeing that the encryption process does not introduce significant overheads or reduce communication efficiency.

To validate the BER performance of the IEAC GCS scheme, the trained GCS is tested in an additive Gaussian noise channel (AWGN) across different signal-to-noise ratios (SNRs) ranging from 6 dB to 20 dB. The comparison with traditional QAM (square constellations) is chosen to demonstrate that integrating encryption via IEAC does not degrade performance relative to widely adopted industry standards. Conventional QAM is the baseline for optical communication systems, and showing parity with it validates that IEAC achieves security without sacrificing the efficiency expected in high-speed networks.

**Figure 3.**
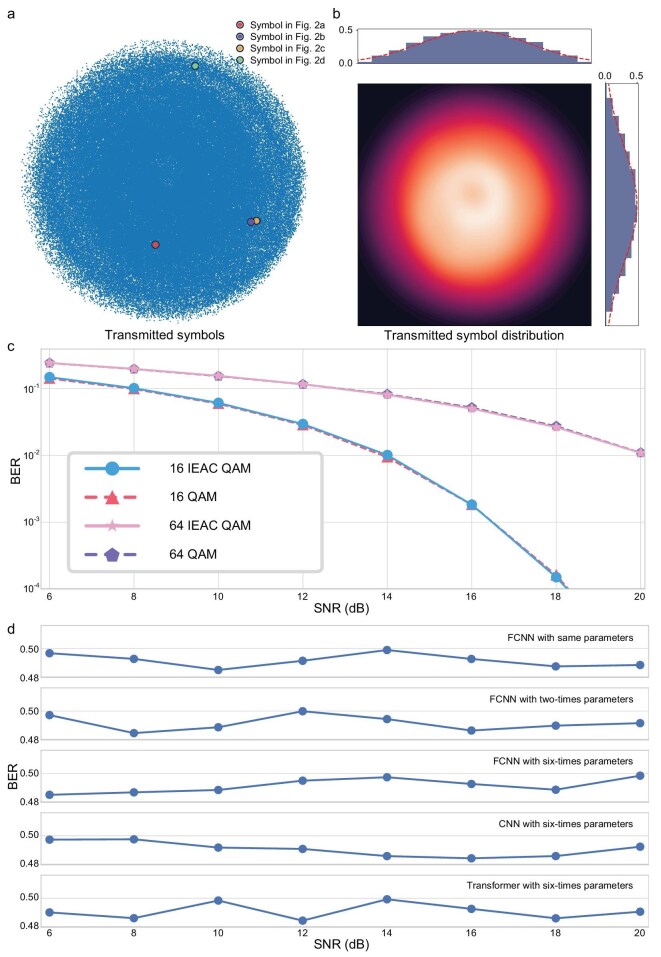
Illustration of GCS scheme within IEAC framework. (a) Representation of transmitted symbols derived from 50 000 random inputs, where the chosen symbols are from Fig. 2a–d in red, purple, orange, and green, respectively. (b) Probability density of transmitted symbols, exhibiting peak concentration at the core and diminishing toward the margin. The marginal distributions along the *x*-axis (top) and *y*-axis (right) feature with Gaussian distribution in the red dashed line. (c) The BER performance with SNR for several constellations: circle solid line represents 16 IEAC QAM, triangle dashed line represents 16 QAM, star solid line represents 64 IEAC QAM, and pentagon dashed line represents 64 QAM. (d) The BER performance with SNR for several decoders with different parameters and structures.

Theoretically, the optimal GCS scheme depends on the SNR, so the SNR is set as a condition and inputted to the encoder with random keys to generate a suitable GCS scheme. After training, different seeds for random keys and information bits are generated to test GCS performance. We use traditional square QAM as the baseline. The results are shown in Fig. 3c, where 16-ary and 64-ary modulations are tested. The trained IEAC QAM performs comparably to traditional QAM in both 16-ary and 64-ary modulation. The encryption, driven by high-speed random keys, ensures that each symbol's GCS is uniquely altered. Even if an eavesdropper employs a decoder architecture distinct from the training, the stochastic nature of the encryption key and dynamic GCS mappings render the intercepted signal statistically indistinguishable from noise.

Here, we evaluate the eavesdropping performance with varying training of the Eve decoder to assess the security, compared to 16-QAM cases in Fig. 3c. The training structure of the Eve decoder is represented by a FCNN, while the testing structures include the same FCNN, a NN with twice the parameters of the training model, and 3 six-times scaled NNs: FCNN, convolutional neural network (CNN), and transformer. All Eve decoders are trained with the true labels without keys. The results, presented in Fig. 3d, show that all bit error rates (BERs) exceed 0.48, indicating that regardless of the NN architecture used, the Eve decoder is unable to effectively decode the information without the keys.

Benefiting from the holistic design for communication and security, the trained GCS scheme maintains communication performance while ensuring robust security. Subsequently, we apply the IEAC GCS scheme in optical fiber communications to achieve high-speed and long-haul secure transmission.

## EXPERIMENTAL DEMONSTRATION OF INTEGRATED ENCRYPTION AND COMMUNICATION IN HIGH-SPEED OPTICAL FIBER TRANSMISSION

**Figure 4.**
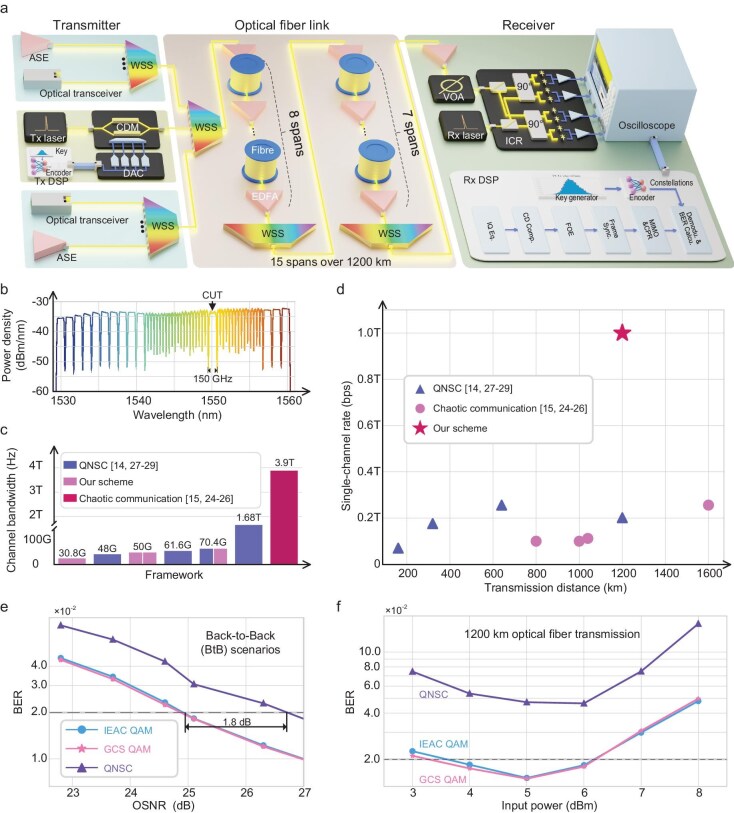
Experimental evaluation of IEAC in a 1 Tb/s WDM long-haul fiber transmission scenario. (a) Depiction of the WDM experimental setup, encompassing 1200 km fiber transmission through 15 spans (80 km each), with a WSS employed for gain flattening in the middle. (b) Identification of the channels under test at 1550 nm within a 26-channel, full C-band WDM transmission. (c) Bar graph illustrating the total channel bandwidth achieved by distinct frameworks, represented by blue (QNSC), purple (chaotic communication), and red (our proposed scheme) bars. (d) Single-channel bit rate plotted against transmission distance for various frameworks, denoted by blue triangles (QNSC), purple circles (chaotic communication), and red stars (our scheme). (e) BtB BER performance at varying OSNR levels. (f) The IEAC's performance after 1200 km of fiber transmission.

The proposed IEAC framework is experimentally validated in a long-haul coherent optical fiber transmission environment, as illustrated in Fig. 4a. The experiment employs 125 GBaud dual-polarization symbols, each carrying 4 bits of input data, generated by the trained encoder (experiment details provided in the Methods section). This configuration yields a single-channel rate of 125 × 4 × 2 = 1 Tb/s. These symbols undergo transmitter-side digital signal processing (DSP, see Supplementary Data for details) and are subsequently modulated via a coherent driver modulator (CDM). A channel spacing of 150 GHz is established using a wavelength selective switch (WSS), culminating in a 26-channel, 3.9 THz bandwidth, full C-band WDM system (depicted in Fig. 4b). Following a 1200 km fiber transmission, the encrypted signals are sampled, subjected to receiver-side DSP, and ultimately decrypted into bits using the correct key.

We benchmark the secure optical communications performance of our scheme against previous works, focusing on chaotic communication and QNSC frameworks. Figure 4c presents the transmission bandwidth utilized in these comparative experiments, while Fig. 4d graphically depicts single-channel rate as a function of transmission distance. Our approach seamlessly integrates with WDM systems, enabling a full C-band transmission—the broadest bandwidth among previous studies. Technologically, it accommodates a larger number of channels, extending coverage to the L band, thereby augmenting overall capacity. Moreover, our experimental demonstration sets a record with a single-channel transmission speed of 1 Tb/s, aligning with the cutting-edge advancements in optical communication technology. This marks the first instance of a system simultaneously achieving wide-bandwidth, high-speed, and long-haul secure transmission.

Figure 4e–f presents the communication performance in our experimental transmission. Here, we present comparisons on the E2EDL-GCS QAM, IEAC QAM, and QNSC schemes. To facilitate validation, we initially trained the IEAC encoder in simulations before deploying it in the actual experiment. Commencing with the back-to-back (BtB) performance assessment in Fig. 4e, we observe that the IEAC OSNR hovers around 25 dB at the forward error correction (FEC) threshold. The results demonstrate that GCS QAM and IEAC QAM exhibit nearly identical performance, while the QNSC scheme shows the worst performance, with a 1.8 dB OSNR degradation. Post a 1200 km optical fiber transmission, both IEAC and GCS QAM BERs remain below 2E-2, reflecting a commendable performance (see Fig. 4f). All BERs initially decrease and then increase with input power, which is due to fiber nonlinearity. At the highest input power, where nonlinearity is significant, IEAC QAM maintains its transmission performance, validating IEAC's robustness in nonlinear-dominated scenarios. Figure 4c-f underscore the unprecedented achievement of record-breaking bit rates for secure WDM transmission, coupled with robust security, over long-haul optical fiber links. Furthermore, with advancements in DSP technology and fine-tuning techniques tailored to real-world scenarios, the IEAC framework holds promise to rival or even surpass conventional communication setups.

**Figure 5.**
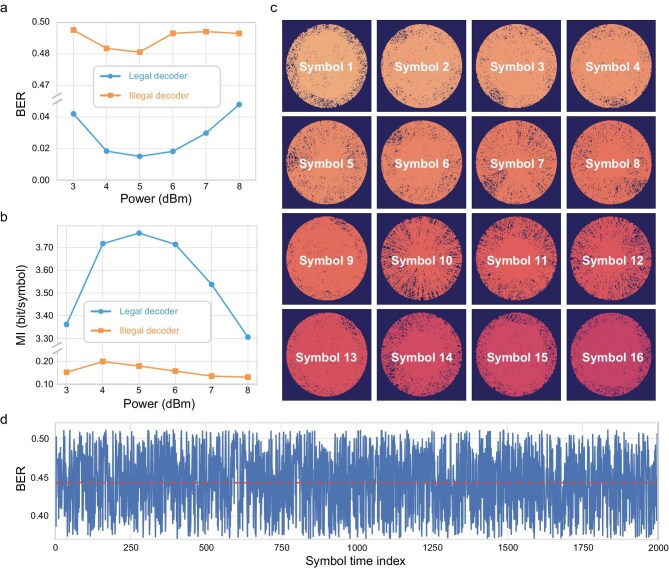
The security performance analysis within IEAC framework. (a) Comparative BER performance of legal users and illegal users. (b) MI analysis for both legal users and illegal users. (c) Temporal evolution of symbol trajectories in the constellation plane under diverse key configurations, demonstrating their inherent disorder. (d) Decoding BER performance utilizing the previous constellation map.

The IEAC framework does not cover the actual signal with noise, and thus cannot be validated through the number of marked symbols (NMS). Instead, IEAC alternates the modulation format for each symbol, rendering the output indistinguishable from noise, as illustrated in Fig. 3a. To assess information leakage, we utilize MI as a more direct cryptographic security indicator. Figure 5a and b exhibit the BER and MI performance for both legal and illegal users. Testing these metrics across 30 consecutive batches, each containing 163 840 bits and with differing bit stream seeds, reveals that the BER and MI for illegal users are consistently around 0.49 and 0.2 bits per symbol, respectively, underscoring the high-security nature of our transmission. Security robustness is further illuminated by examining the trajectory of each symbol on a two-dimensional plane, with the encryption key updated 2000 times. Figure 5c demonstrates that the path traced by each symbol is highly chaotic and disorderly, rendering any discernible pattern that could be exploited for decryption purposes effectively obscured due to the substantial variations introduced between successive mappings. We further evaluate the BER performance under the assumption that illegal users could potentially access the GCS map from the previous transmission for decoding subsequent communications. As demonstrated in Fig. 5d, even when illegal users employ the last known GCS map for decoding, the BER remains high at around 0.45. This is a direct consequence of the IEAC system employed, where each key update significantly alters the GCS pattern. Such a strategy ensures that even if an eavesdropper could somehow track the symbol's movement over time, the erratic and unpredictable nature of these maps would avoid the derivation of any meaningful information. This outcome underscores the robustness of our IEAC system in maintaining security and communication performance of transmitted data against sophisticated attack scenarios.

The IEAC framework maximizes MI for legitimate users through adversarial training, where the encoder and legal decoder (Bob) are co-optimized using cross-entropy loss (Fig. 1b). To further improve MI, one can incorporate reinforcement learning to refine the encoder's ability to prioritize MI maximization under varying channel conditions. Additionally, advanced training strategies, such as warmup training, could be explored to strengthen the adversarial training process. Moreover, further investigation into online training with real channel data will be conducted to enhance MI performance, particularly in nonlinear, low-SNR environments.

The IEAC framework, based on E2EDL, is designed with flexibility and scalability, making it applicable to diverse communication environments, such as SDM channels [47,48]. The IEAC framework's neural networks (encoder/decoder) can be reconfigured to process spatial channels by expanding input/output dimensions and incorporating spatial channel models during training. While our current experiments focus on SMF/WDM systems, extending IEAC to SDM and other channels is a promising direction. Future studies could leverage the same adversarial training principles to jointly optimize spatial and spectral efficiency.

## DISCUSSION

Our work provides a foundational step towards realizing the full potential of optical fiber communications in the era of AI and big data. By successfully integrating E2EDL with GCS for encryption, we have demonstrated a scalable and efficient solution to the long-standing challenge of securing high-speed long-haul data transmissions. The successful demonstration of a single-channel 1 Tb/s secure transmission over 1200 km, concurrently with a full C-band transmission, represents a monumental advancement in the field of optical secure communications. This achievement not only facilitates E2EDL training through backpropagation to enhance data transmission security but also underscores the viability of GCS in optimizing encryption while maintaining communication performance. The IEAC framework can be extended to diverse communication environments with suitable channel modeling and DSP techniques, such as SDM channels. The implications of this work extend beyond the telecommunications industry, offering insights into the design of future secure, high-capacity networks capable of supporting the burgeoning demands of global data traffic and AI computation.

## METHODS

### Key distribution mechanism

In the IEAC framework, QKD is employed to securely distribute the initial seed key between the sender (Alice) and receiver (Bob) [49]. The seed is input into a cryptographically secure pseudo-random number generator (e.g. Permuted Congruential Generator, PCG) to produce a long-period key stream. This stream dynamically selects GCS mappings for each symbol, enabling one-time pad-like encryption. During this processing, the prior knowledge of the key is essential for legitimate users. The legal decoder relies on the key to dynamically reconfigure its GCS mappings (Fig. 2) and invert the encryption process. Without the key, the received symbols appear as noise (Fig. 5c), and MI for illegal users remains near zero (Fig. 5b).

To mitigate risks of key exhaustion or potential leaks, the seed key is periodically refreshed via QKD. This ensures continuous security without interrupting transmission. The period can be determined based on the QKD and information transmission rate.

While QKD is referenced as a potential key distribution method, the current implementation employs a pre-shared key approach for simplicity and practicality in experimental validation. Validation of the joint system's performance in a 1200 km testbed can be investigated in the future, measuring key refresh rates and their impact on security.

### The experiment setup for the IEAC framework

As depicted in Fig. 4a, the experimental setup for the IEAC framework revolves around a WDM system. In the transmitter, 125 GBaud dual-polarization symbols are generated, shaped with a root-raised cosine filter featuring a 0.1 roll-off factor, pre-compensated for bandwidth, quantized into 256 levels, and subsequently fed to a DAC operating at a 175 GSample/s rate. Post-DAC, the signals are optically modulated via a CDM. The combined bandwidth of the DAC and CDM amounts to 75 GHz.

To realize a full C-band WDM transmission, we employ a two-stage multiplexing strategy WSS. The first-stage WSS combines the 25 individual WDM channels, while the second-stage WSS integrates the channel under test (CUT) with the remaining channels. Additionally, the second-stage WSS performs power allocation to mitigate power imbalances along the fiber links. As shown in Fig. 4b, three 50G optical transceivers are concatenated into a single channel with a 150 GHz spacing, which are placed on both sides of the CUT. The remaining channels are filled with amplified spontaneous emission (ASE) noise. The CUT is set at 1550 nm in the experiment.

The 1200 km fiber link comprises 15 spans of standard single-mode fiber (SSMF) interspersed with Erbium-Doped fiber amplifiers (EDFAs) exhibiting a 5 dB noise figure. After traversing 8 fiber spans, another WSS is employed for power allocation in the remaining fiber spans. Following the entire 15-span transmission, the CUT is demultiplexed using another WSS and EDFA. The received signals are then captured by an integrated coherent receiver (ICR) and an oscilloscope, both equipped with a 110 GHz bandwidth. Then the advanced DSP algorithms must be adapted to account for the dynamic modulation characteristics in order to compensate for channel distortions [50].

## Supplementary Material

nwaf112_Supplemental_File

## References

[1] Xu L, Jiang C, Wang J et al. Information security in big data: privacy and data mining. IEEE Access 2014; 2: 1149–76.

[2] Bertino E, Kantarcioglu M, Akcora CG et al. AI for security and security for AI. In: Proceedings of the Seventh ACM On Conference on Data and Application Security and Privacy. New York, USA, 22–24 March 2021.

[3] Li J-h . Cyber security meets artificial intelligence: a survey. Front Inform Tech El 2018; 19: 1462–74.

[4] Fok MP, Wang Z, Deng Y et al. Optical layer security in fiber-optic networks. IEEE Trans Inf Forensics Secur 2011; 6: 725–36.

[5] Guan K, Cho J, Winzer PJ. Physical layer security in fiber-optic MIMO-SDM systems: an overview. Opt Commun 2018; 408: 31–41.

[6] Liu Y, Chen H-H, Wang L. Physical layer security for next-generation wireless networks: theories, technologies, and challenges. IEEE Commun Surv Tut 2016; 19: 347–76.

[7] Song H, Lin R, Li Y et al. Machine-learning-based method for fiber-bending eavesdropping detection. Opt Lett 2023; 48: 3183–6. 37319057 10.1364/OL.487214

[8] Liu T, Wang W, Ouyang F et al. Eavesdropping-aware survivable routing in physical-layer secured optical networks. J Opt Commun Netw 2025; 17: 127–38.

[9] Bloch M, Barros J. Physical-layer Security: From Information Theory to Security Engineering. Cambridge: Cambridge University Press, 2011.

[10] Sun H, Torbatian M, Karimi M et al. 800G DSP ASIC design using probabilistic shaping and digital sub-carrier multiplexing. J Light Technol 2020; 38: 4744–56. 10.1109/JLT.2020.2996188

[11] Zhou YR, Keens J, Wakim W. High capacity innovations enabling scalable optical transmission networks. J Light Technol 2022; 41: 957–67. 10.1109/JLT.2022.3206277

[12] Zhang D, Zuo M, Chen H et al. Technological prospection and requirements of 800G transmission systems for ultra-long-haul all-pptical terrestrial backbone networks. J Light Technol 2023; 41: 3774–82. 10.1109/JLT.2023.3267241

[13] Luo W, Cao L, Shi Y et al. Recent progress in quantum photonic chips for quantum communication and internet. Light Sci Appl 2023; 12: 175. 10.1038/s41377-023-01173-8 37443095 PMC10345093

[14] Sun J, Jiang L, Yi A et al. Experimental demonstration of 201.6-gbit/s coherent probabilistic shaping QAM transmission with quantum noise stream cipher over a 1200-km standard single mode fiber. Opt Express 2023; 31: 11344–53. 10.1364/OE.484431 37155772

[15] Feng J, Jiang L, Sun J et al. 256 Gbit/s chaotic optical communication over 1600 km using an AI-based optoelectronic oscillator model. J Light Technol 2024; 42: 2774–83. 10.1109/JLT.2024.3352892

[16] Gisin N, Thew R. Quantum communication. Nat Photon 2007; 1: 165–71. 10.1038/nphoton.2007.22

[17] Nauerth S, Moll F, Rau M et al. Air-to-ground quantum communication. Nat Photon 2013; 7: 382–6. 10.1038/nphoton.2013.46

[18] Bhaskar MK, Riedinger R, Machielse B et al. Experimental demonstration of memory-enhanced quantum communication. Nature 2020; 580: 60–4. 10.1038/s41586-020-2103-5 32238931

[19] Li W, Zhang L, Tan H et al. High-rate quantum key distribution exceeding 110 mb s^–1^. Nat Photon 2023; 17: 416–21. 10.1038/s41566-023-01166-4

[20] Yang J, Jiang Z, Benthin F et al. High-rate intercity quantum key distribution with a semiconductor single-photon source. Light Sci Appl 2024; 13: 150. 10.1038/s41377-024-01488-0 38956020 PMC11219984

[21] Pirandola S, Laurenza R, Ottaviani C et al. Fundamental limits of repeaterless quantum communications. Nat Commun 2017; 8: 15043. 10.1038/ncomms15043 28443624 PMC5414096

[22] Argyris A, Syvridis D, Larger L et al. Chaos-based communications at high bit rates using commercial fibre-optic links. Nature 2005; 438: 343–6. 10.1038/nature04275 16292256

[23] Yang Z, Yi L, Ke J et al. Chaotic optical communication over 1000 km transmission by coherent detection. J Light Technol 2020; 38: 4648–55. 10.1109/JLT.2020.2994155

[24] Jiang L, Pan Y, Yi A et al. Trading off security and practicability to explore high-speed and long-haul chaotic optical communication. Opt Express 2021; 29: 12750–62. 10.1364/OE.423098 33985025

[25] Xie Y, Yang Z, Shi M et al. 100 Gb/s coherent chaotic optical communication over 800 km fiber transmission via advanced digital signal processing. Adv Photonics Nexus 2024; 3: 016003.

[26] Wu Y, Zhang Z, Luo H et al. 100Gb/s coherent optical secure communication over 1000 km based on analog-digital hybrid chaos. Opt Express 2023; 31: 33200–11. 10.1364/OE.499634 37859105

[27] Yoshida M, Hirooka T, Kasai K et al. Single-channel 40 gbit/s digital coherent QAM quantum noise stream cipher transmission over 480 km. Opt Express 2016; 24: 652–61. 10.1364/OE.24.000652 26832295

[28] Chen X, Tanizawa K, Winzer P et al. Experimental demonstration of a 4,294,967,296-QAM-based Y-00 quantum stream cipher template carrying 160-gb/s 16-QAM signals. Opt Express 2021; 29: 5658–64. 10.1364/OE.405390 33726100

[29] Li Y, Li Y, Zhu K et al. Analysis of the encryption penalty in a QAM-based quantum noise stream cipher. Opt Express 2023; 31: 19006–20. 10.1364/OE.489043 37381327

[30] Barber D, Agakov F. The IM algorithm: a variational approach to information maximization. In: Proceedings of the 17th International Conference on Neural Information Processing Systems. Whistler BC, Canada, 9–11 December 2003.

[31] Poole B, Ozair S, Van Den Oord A et al. On variational bounds of mutual information. In: Proceedings of the 36th International Conference on Machine Learning. California, USA, 9–15 June 2019.

[32] Karanov B, Lavery D, Bayvel P et al. End-to-end optimized transmission over dispersive intensity-modulated channels using bidirectional recurrent neural networks. Opt Express 2019; 27: 19650–63. 10.1364/OE.27.019650 31503722

[33] Stark M, Aoudia FA, Hoydis J. Joint learning of geometric and probabilistic constellation shaping. 2019 IEEE Globecom Workshops. Waikoloa, HI, USA, 9–13 December 2019.

[34] Niu Z, Yang H, Zhao H et al. End-to-end deep learning for long-haul fiber transmission using differentiable surrogate channel. J Lightwave Technol 2022; 40: 2807–22. 10.1109/JLT.2022.3148270

[35] Belghazi MI, Baratin A, Rajeshwar S et al. Mutual information neural estimation. In: Proceedings of the 35th International Conference on Machine Learning. Stockholm, Sweden, 10–15 July 2018.

[36] Niu Z, Dai C, Yang H et al. Enhanced mutual information neural estimators for optical fiber communication. Opt Lett 2024; 49: 4381–4. 10.1364/OL.534025 39090938

[37] Goodfellow I, Pouget-Abadie J, Mirza M et al. Generative adversarial nets. In: Proceedings of the 28th International Conference on Neural Information Processing Systems. Montreal, Canada, 8–13 December 2014.

[38] Jovanovic O, Da Ros F, Zibar D et al. Geometric constellation shaping for fiber-optic channels via end-to-end learning. J Light Technol 2023; 41: 3726–36. 10.1109/JLT.2023.3276300

[39] Neskorniuk V, Carnio A, Bajaj V et al. End-to-end deep learning of long-haul coherent optical fiber communications via regular perturbation model. 2021 European Conference on Optical Communication. Bordeaux, France, 13–16 September 2021.

[40] Rumelhart DE, Hinton GE, Williams RJ. Learning representations by back-propagating errors. Nature 1986; 323: 533–6. 10.1038/323533a0

[41] Wang D, Song Y, Li J et al. Data-driven optical fiber channel modeling: a deep learning approach. J Light Technol 2020; 38: 4730–43. 10.1109/JLT.2020.2993271

[42] Yang H, Niu Z, Xiao S et al. Fast and accurate optical fiber channel modeling using generative adversarial network. J Lightwave Technol 2021; 39: 1322–33. 10.1109/JLT.2020.3037905

[43] Dzieciol H, Liga G, Sillekens E et al. Geometric shaping of 2-D constellations in the presence of laser phase noise. J Light Technol 2020; 39: 481–90. 10.1109/JLT.2020.3031017

[44] Mirani A, Agrell E, Karlsson M. Low-complexity geometric shaping. J Light Technol 2020; 39: 363–71. 10.1109/JLT.2020.3033031

[45] Sillekens E, Liga G, Lavery D et al. High-cardinality geometrical constellation shaping for the nonlinear fibre channel. J Light Technol 2022; 40: 6374–87. 10.1109/JLT.2022.3197366

[46] Harris CR, Millman KJ, Van Der Walt SJ et al. Array programming with NumPy. Nature 2020; 585: 357–62. 10.1038/s41586-020-2649-2 32939066 PMC7759461

[47] Xu Q, Gao R, Wang F et al. End-to-end learning of joint noise shaping and probabilistic shaping for OAM mode division multiplexing transmission. Opt Lett 2024; 49: 5767–70. 10.1364/OL.535092 39404533

[48] Rothe S, Besser K-L, Krause D et al. Securing data in multimode fibers by exploiting mode-dependent light propagation effects. Research 2023; 6: 0065. 10.34133/research.0065 36930761 PMC10013962

[49] Huang X, Peng X, Zhang L et al. Coexistence of high-speed physical-layer key distribution and secure data transmission in fiber. J Lightwave Technol 2023; 42: 572–8. 10.1109/JLT.2023.3316270

[50] Niu Z, Yang H, Li L et al. Learnable digital signal processing: a new benchmark of linearity compensation for optical fiber communications. Light Sci Appl 2024; 13: 188. 10.1038/s41377-024-01556-5 39134543 PMC11319808

