# Supplementary material for: Experimental demonstration of integrated encryption and communication over optical fiber
Source: Natl Sci Rev. 2025 Apr 2;12(7):nwaf112. doi: 10.1093/nsr/nwaf112 (PMC12153715; doi:10.1093/nsr/nwaf112)
Supplement: nwaf112_Supplemental_File [file nwaf112_supplemental_file.docx]

**Supplementary File for Experimental Demonstration of Integrated Encryption and Communication over Optical Fibre**

**Zekun Niu, Yunhao Xie, Guozhi Xu, Chenhao Dai, Hang Yang, Chuyan Zeng, Minghui Shi, Lyv Li, Guoqing Pu, Weisheng Hu & Lilin Yi^*^**

State Key Laboratory of Photonics and Communications, School of Electronic Information and Electrical Engineering, Shanghai Jiao Tong University, Shanghai 200240, China.

Corresponding author: **^*^**lilinyi@sjtu.edu.cn

**Mutual information neural estimation**

Due to the fibre nonlinearities (e.g., Kerr effect, cross-phase modulation) distort signal statistics, traditional MI estimation methods based on the Gaussian approximations might be inaccurate. To address this problem, MINE is utilized for estimating and optimizing the MI for illegal users in the IEAC framework. Given two random variables ***X*** and ***Y***, whose realizations are ***x*** and ***y*** respectively, the MI can be mathematically expressed by Donsker-Varadhan representation, which is an approximation of relative entropy [1]:

where sup is the supremum taken over all functions *T* within a class of functions *T_θ_*, ensuring that the expectations in Eq. (S1) are finite. Indeed, the function sets *T_θ_* can be realized by a neural network with parameters *θ*. In Equation (S1), both the left and right components represent expectations based on samples drawn from the joint and marginal probability distributions, respectively. MINE’s model-agnostic, data-driven approach is suitable for securing high-speed optical communications in realistic, nonlinear channels.

In practical implementation, Eq. (S1) can be treated as a loss function, allowing the MI to be estimated via a stochastic gradient ascent algorithm. The sampling necessary for this estimation is derived from a mixture of received signals, as illustrated in Figure 1c in the main text. The training process involves initially training the MINE, followed by incorporating it into the loss function given in Eq. (S1) to facilitate the security-enhancing training of the encoder. By training alongside the encoder and decoder neural networks, MINE allows dynamic adaptation to channel conditions. This co-optimization ensures MI minimization for eavesdroppers remains robust under varying impairments.

We denote that the accuracy and applicability are investigated in our previous work [2]. One can find more information about MINE training and implementation.

**End-to-end deep learning training strategy for integrated encryption and communication.**

The E2EDL training unfolds in three steps to ensure comprehensive learning and optimization of the IEAC. Initially, the DSC and decoders are trained according to the transmission results. The DSC are trained as a regression task employing a Mean Square Error (MSE) loss function. Concurrently, decoders for both legal and illegal receivers are trained using the known plaintext data. The critical distinction between these two decoders lies in the input of the encryption key to legal decoders, absent in the training of illegal decoders. Subsequently, the MINE is trained to accurately estimate the MI between the ordinary signal before encryption and the received signals without the key. The final stage of the training process focuses on the encoder, where the goal is to optimize the signal's encryption and communication performance based on Eq. (1) in the main text. It should be noted that the loss in Eq. (1) is applied to bits, thereby transforming the CE and MI losses into Binary Cross Entropy (BCE) loss and Generalized Mutual Information (GMI), respectively.

In the above three steps, a strategy involving an exponential increase in the number of keys is employed to mitigate the risk of training failures resulting from an abundance of random keys. During the early stages of training, our objective is to induce the encoder to establish secure coding rules to enhance overall security. In the later stages of training, as the growing number of keys provides sufficient security, our focus shifts towards improving communication quality. Consequently, an exponential decay factor is incorporated into the ${Loss}_{MINE}$ term in Eq. (1) in the main text. This dynamic adjustment influences the relative magnitudes of ${Loss}_{MINE}$ and ${Loss}_{CE}$, guiding the encoder towards optimal training outcomes. This strategic optimization ensures that the E2EDL-IEAC framework achieves its dual objectives: securing data against illegal access and maintaining high capacity in data transmission.

We denote that the training process and trained models do not require secrecy. The adversarial training phase involves simulating eavesdroppers (Eve decoders) to optimize MI bounds. However, the trained model’s security stems from the stochastic key-driven GCS mappings, not the training data or methodology. Even if the training process is public or the models are obtained, an attacker cannot exploit it to derive the key or decode signals, as the key is dynamically refreshed and never exposed during training.

**Digital signal processing for experimental optical fibre secure transmission.**

Upon completion of sampling, DSP on the receiver's side is employed to rectify signal distortions inherent in fibre links, as illustrated in Fig. S1a. The employment of GCS within the IEAC framework necessitates a constellation-independent DSP approach. Existing DSP techniques (e.g., carrier phase recovery (CPR), frequency offset estimation (FOE)) are optimized for static constellations, such as QAM modulation. However, for the IEAC framework, advanced DSP algorithms must be adapted to account for the dynamic modulation characteristics to compensate for channel distortions:

- Pilot-Assisted DSP. To compensate for optical fiber channel distortions, the DSP needs to be modulation-agnostic. In this context, pilot-assisted DSP should be employed to track dynamic distortions such as DGD, LOFO, and phase noise.
- Frame Synchronization. To effectively use the preset pilot symbols, frame synchronization must be established before performing DSP. In long-haul transmission, chromatic dispersion and LOFO can affect synchronization accuracy. Therefore, precise framing methods are essential in this framework.

In our experiment, we use fixed Quadrature Phase Shift Keying (QPSK) as pilot symbols, which are inserted into the data stream (Fig. S1b) to enable FOE, frame synchronization, and initial equalization. These pilots act as stable reference points, decoupling dynamic GCS from critical DSP steps. It is noted that a real modulation format-independent DSP, crucial for both performance and efficiency enhancements through pilot elimination, presents a promising avenue for future investigation. The pilot-based DSP methodology encompasses front-end processing, chromatic dispersion (CD) compensation, FOE, frame synchronization, pilot symbols equalization, and CPR. Initial front-end processing filters signals and corrects IQ impairments, succeeded by frequency domain CD compensation. Subsequently, FOE and frame synchronization processes are executed, culminating in pilot symbols equalization and CPR.


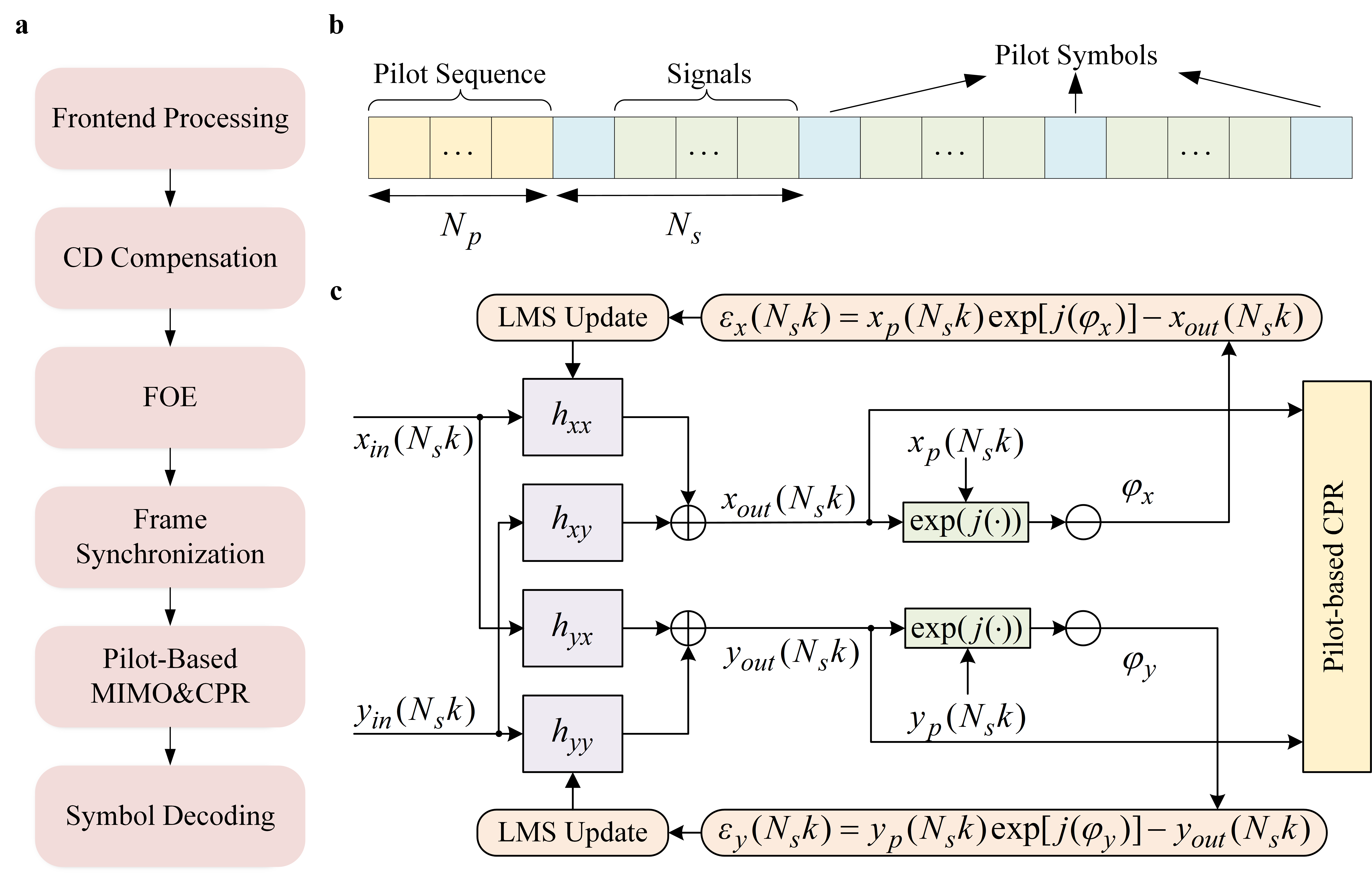


Figure S1 | Receiver-side DSP architecture prior to IEAC symbol decoding in experimental setup. (a) Comprehensive DSP block diagram. (b) Signal frame structure representation. (c) MIMO and CPR configuration for adaptive equalization of polarization-dependent effects and phase noise.

The transmitter's data frame configuration is depicted in Fig. S1b. It begins with a pilot sequence of length $N_{p}$, integrated at the start of the encrypted symbol stream and interspersed with pilot symbols at a ratio of $1/{N_{s}}$. This pilot sequence is designed for frame detection, FOE, and equalization, while the pilot symbols facilitate joint equalization and phase noise correction.

Figure S1c details the operation of the joint equalization and CPR within the pilot-based 2x2 Multiple-Input Multiple-Output (MIMO) equalizer, where $h_{xx}$, $h_{xy}$, $h_{yx}$, $h_{yy}$ represent the MIMO equalizer’s tap coefficients. The input symbols post-frame synchronization, $x_{in}(N_{s}k)$ and $y_{in}(N_{s}k)$, undergo processing. The Least Mean Square (LMS) algorithm, guided by the pilot sequence, drives the equalizer towards convergence. Comparison of pilot symbols $x_{P}(N_{s}k)$, $y_{p}(N_{s}k)$ with the equalized symbols $x_{out}(N_{s}k)$, $y_{out}(N_{s}k)$ allows for the estimation of the current phase $\varphi_{x}$ and $\varphi_{y}$, subsequently adjusting the LMS equalizer's error function:

Upon convergence, the equalizer adeptly tracks fibre channel random effects, such as polarization-dependent effects, across the symbol stream, with tap coefficient adjustments confined to pilot symbol intervals.

**References**

1. Belghazi MI, Baratin A and Rajeshwar S et al. Mutual information neural estimation. Proceedings of the 35th International Conference on Machine Learning, Stockholm, Sweden, 10-15 July 2018.
2. Niu Z, Dai C and Yang H et al. Enhanced mutual information neural estimators for optical fiber communication. Opt Lett 2024; 49: 4381-4384.
